# Fasciculoventricular Pathway Mimicking Wolff-Parkinson-White Syndrome: A Delta Wave That Should Not Be Ablated

**DOI:** 10.1016/j.jaccas.2026.107529

**Published:** 2026-05-20

**Authors:** Takahiro Hayashi

**Affiliations:** Department of Cardiology, NewHeart Watanabe Institute, Tokyo, Japan

**Keywords:** ablation, palpitations, supraventricular arrhythmias

## Abstract

**Background:**

Fasciculoventricular pathways (FVPs) are rare and benign variants of ventricular preexcitation that may closely mimic septal atrioventricular accessory pathways on surface electrocardiography.

**Case Summary:**

A woman in her 40s was referred for suspected Wolff-Parkinson-White syndrome with palpitations. The electrocardiogram demonstrated a short PR interval with subtle preexcitation. Electrophysiological study revealed a fixed His-ventricular interval of 28 milliseconds across sinus rhythm, atrial incremental, and extrastimulus pacing and junctional rhythm. Atrial extrastimulus pacing resulted in disappearance of preexcitation with transition to complete right bundle branch block morphology, reflecting functional right bundle branch refractoriness. These findings were diagnostic of an FVP.

**Discussion:**

A fixed His-ventricular interval across multiple pacing maneuvers represents the key electrophysiological feature distinguishing FVPs from true septal accessory pathways.

**Take-Home Message:**

Accurate recognition of FVPs is essential to avoid misdiagnosis and unnecessary ablation near the His-Purkinje system.

## Visual Summary

**Figure:**
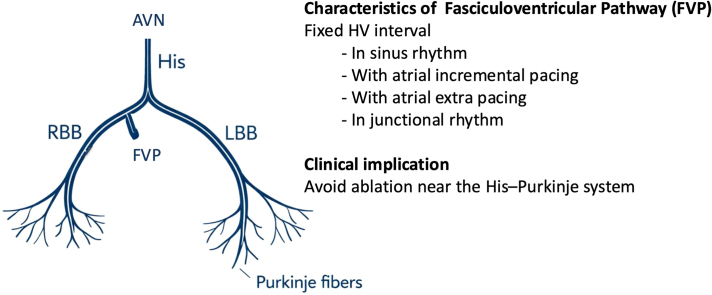

**Visual Summary:**
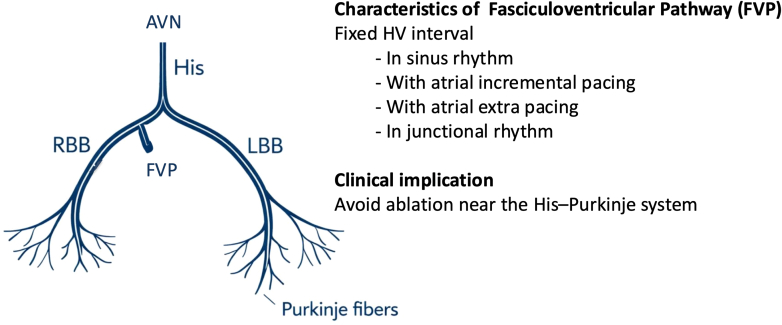
FVP Mimicking Wolff-Parkinson-White Syndrome The key electrocardiographic and electrophysiological features of an FVP are summarized. Despite subtle ventricular preexcitation on surface electrocardiography, a fixed HV interval is consistently observed during sinus rhythm, atrial pacing, atrial extrastimulus pacing, and junctional rhythm. Recognition of this benign pathway is essential to avoid unnecessary ablation near the His-Purkinje system. AVN = Atrioventricular Node; FVP = fasciculoventricular pathway; HV = His-ventricular LBB = Left Bundle Branch; RBB = Right Bundle Branch.

Fasciculoventricular pathways (FVPs) are rare and benign variants of ventricular preexcitation that result from direct connections between the His bundle or proximal fascicles and the ventricular myocardium. Unlike typical atrioventricular accessory pathways, FVPs do not participate in reentrant tachycardias and are generally considered innocent bystanders.^1^^,^^2^

**Take-Home Messages**

• A fixed HV interval across multiple pacing maneuvers is diagnostic of a fasciculoventricular pathway and distinguishes it from true atrioventricular accessory pathways.
• Recognition of this benign form of preexcitation is essential to avoid unnecessary and potentially harmful ablation near the His-Purkinje system.

On surface electrocardiography (ECG), FVPs may present with subtle delta waves, a short PR interval, and a near-normal QRS duration, often mimicking septal accessory pathway–mediated pre-excitation.^1^^,^^2^ As a result, patients with FVPs are frequently referred for invasive electrophysiological evaluation with suspected Wolff-Parkinson-White (WPW) syndrome.

Accurate recognition of this entity is clinically important because ablation of an FVP is unnecessary and may pose a risk of atrioventricular block due to its close proximity to the His-Purkinje system.^3^ We report a case of an FVP presenting with subtle preexcitation that closely mimicked WPW syndrome, highlighting key electrocardiographic and electrophysiological features that help avoid inappropriate ablation.

## Case Presentation

A woman in her 40s was referred to our institution after an abnormal ECG detected during a routine health checkup suggested WPW syndrome. She reported occasional palpitations but denied syncope or documented tachyarrhythmias. There was no family history of sudden cardiac death.

Given the presence of symptoms and suspected ventricular preexcitation, an electrophysiological study was performed.

## ECG Findings (ECG Challenge)

The baseline 12-lead ECG demonstrated a short PR interval of 115 milliseconds with subtle ventricular preexcitation. The QRS complex was isoelectric in lead V_1_, whereas small positive delta waves were observed in leads V_5_ and V_6_ (Figure 1). The overall QRS duration was near normal.

**Figure 1:**
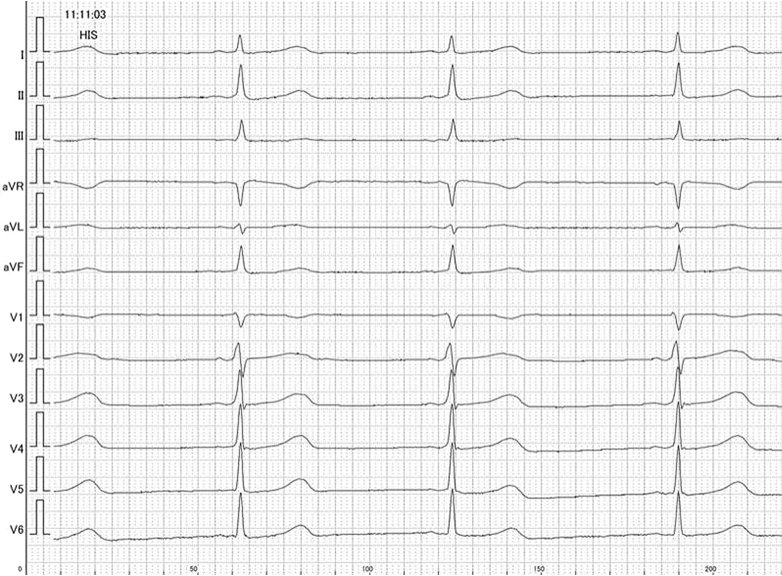
Baseline 12-Lead Electrocardiogram Baseline sinus rhythm demonstrating a short PR interval (115 milliseconds) with subtle ventricular preexcitation. The QRS complex is isoelectric in lead V_1_, with small positive delta waves in leads V_5_ and V_6_. Overall QRS duration is near normal.

These findings raised suspicion for a septal accessory pathway.

What is the most likely mechanism of ventricular preexcitation in this patient?

## Electrophysiological Study

On admission, vital signs were within normal limits: blood pressure was 106/62 mm Hg; heart rate was 70 beats/min; oxygen saturation was 98% on room air and body temperature within the normal range. Physical examination was unremarkable, with no cardiac murmurs, no signs of heart failure, and normal respiratory findings.

Intracardiac recordings revealed the following:

• Baseline His-ventricular (HV) interval was 28 milliseconds (Figure 2).

**Figure 2:**
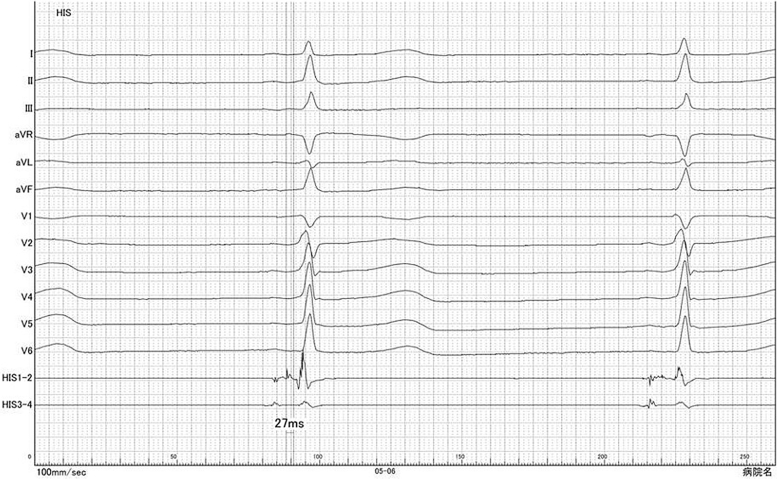
Baseline His-Ventricular Interval Intracardiac electrograms during sinus rhythm demonstrating a fixed His-ventricular interval of 28 milliseconds.
• During incremental atrial pacing, the HV interval remained unchanged despite increasing atrial rates (Figure 3).

**Figure 3:**
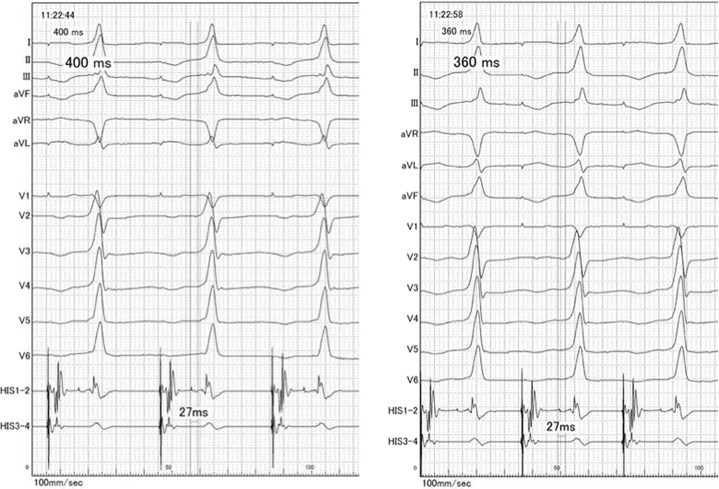
Fixed His-Ventricular Interval With Decremental Atrial Pacing Intracardiac recordings during atrial pacing with 400-millisecond (left panel) and 360-millisecond (right panel) pacing rates. Despite increasing atrial rates, the His-ventricular interval remains unchanged, consistent with a fasciculoventricular pathway.
• Ventriculoatrial conduction was present and decremental.
• Atrial extrastimulus pacing demonstrated atrial-His decrementing without disappearance of ventricular preexcitation (Figure 4).

**Figure 4:**
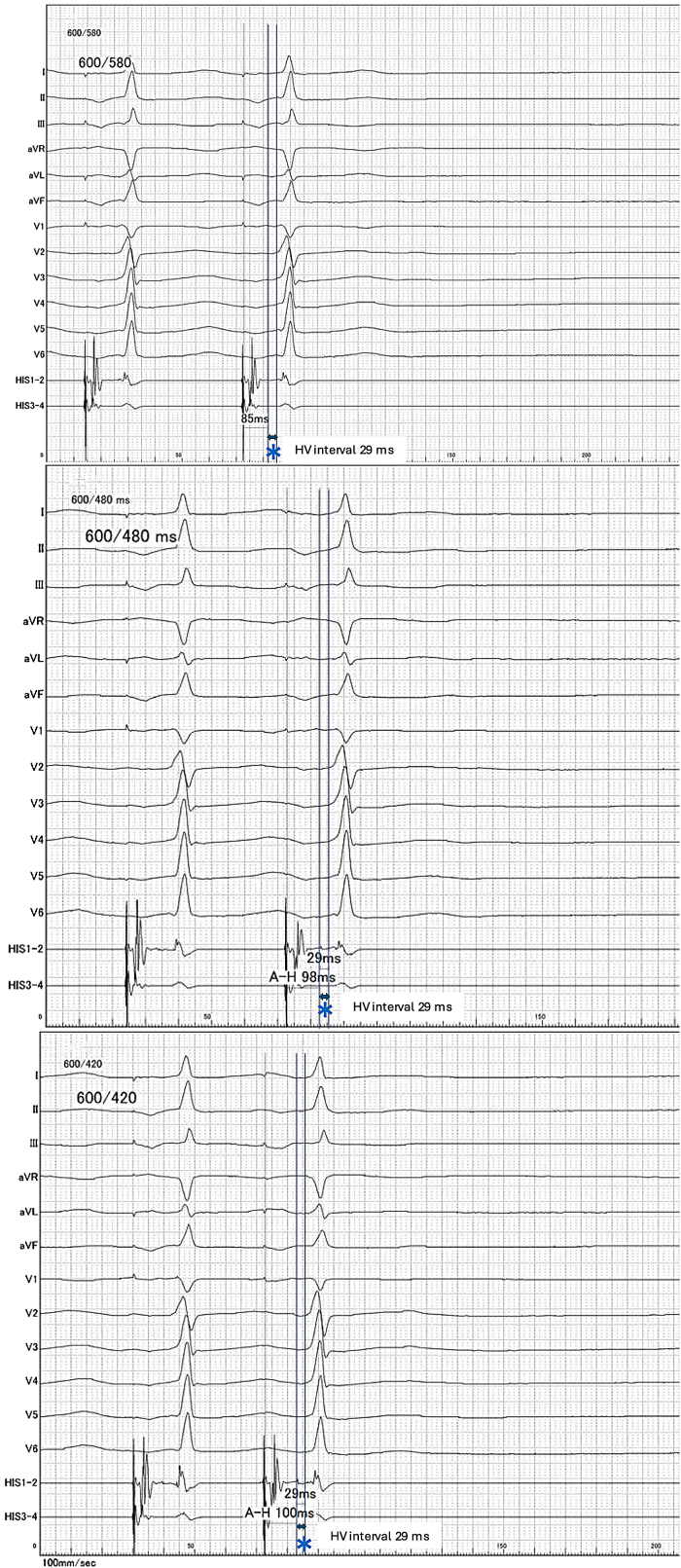
Atrial Extrastimulus Testing Demonstrating atrial-His Decrement With Fixed His-Ventricular Interval Atrial extrastimulus pacing (600/580, 600/480, and 600/420 milliseconds) demonstrating progressive atrial-His prolongation without disappearance of ventricular preexcitation, indicating atrioventricular nodal decremental conduction. Blue asterisks mark the His-ventricular interval, which remained constant at 29 milliseconds during all pacing maneuvers.
• At an atrial pacing cycle length of 600/400 milliseconds, ventricular preexcitation disappeared and the QRS complex transitioned to a complete right bundle branch block morphology (Figure 5).

**Figure 5:**
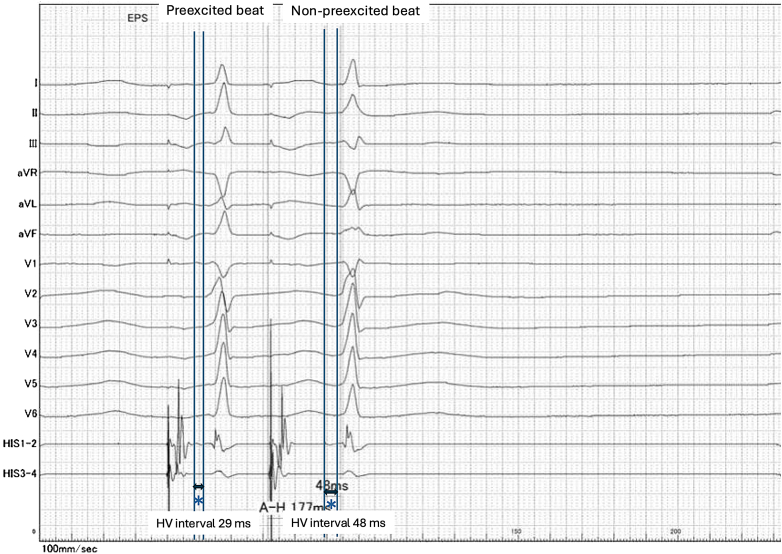
Loss of Preexcitation With Emergence of Complete Right Bundle Branch Block At an atrial pacing cycle length of 600/400 milliseconds, ventricular preexcitation disappears and the QRS complex transitions to a complete right bundle branch block morphology, reflecting functional right bundle branch refractoriness. Asterisks mark the HV interval for each beat. The HV interval measures 29 milliseconds in the preexcited beat and 48 milliseconds in the non-preexcited beat. In the preexcited beat, delta waves are most prominent in leads V_5_ and V_6_. HV = His-ventricular.
• During junctional rhythm, the HV interval remained constant at 28 milliseconds (Figure 6).

**Figure 6:**
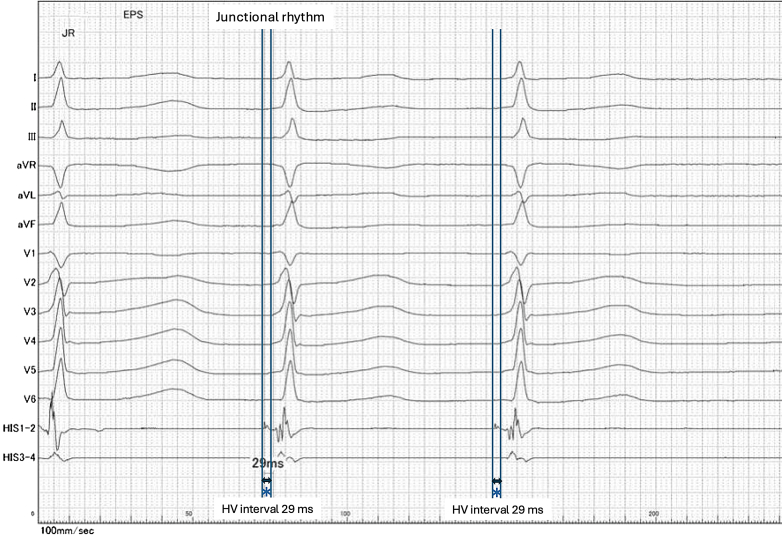
Fixed His-Ventricular Interval During Junctional Rhythm Intracardiac recordings during junctional rhythm demonstrating a persistently fixed His-ventricular interval of 29 milliseconds, further supporting the diagnosis of a fasciculoventricular pathway. Asterisks mark the His-ventricular interval for each beat.
• Isoproterenol infusion revealed dual atrioventricular nodal physiology, but the degree of ventricular preexcitation was unchanged.
• No supraventricular or ventricular tachycardia was inducible.

## Diagnosis

• The constellation of electrophysiological findings was inconsistent with a typical atrioventricular accessory pathway. In particular, the fixed HV interval across sinus rhythm, atrial incremental and extrastimulus pacing, and junctional rhythm strongly argued against an atrioventricular bypass tract.
• The disappearance of preexcitation with atrial extrastimulus pacing and the emergence of complete right bundle branch block morphology reflected functional refractoriness of the right bundle branch, rather than loss of accessory pathway conduction.
• These findings are diagnostic of an FVP.

## Management and Clinical Course

Although the FVP itself was considered benign and unlikely to account for the patient's palpitations, dual atrioventricular nodal physiology was demonstrated during isoproterenol infusion. Given the patient's occasional palpitation symptoms, slow pathway ablation was performed.

No ablation targeting the FVP was attempted. The patient had an uneventful postprocedural course and was discharged the following day.

## Discussion

FVPs represent a distinct and uncommon cause of ventricular pre-excitation, characterized by direct conduction from the His bundle or proximal fascicles to the ventricular myocardium.^1^^,^^4^ Because these pathways bypass only a short segment of the normal conduction system, they do not support atrioventricular reentrant tachycardia and are typically benign.^1^^,^^2^ Previous case reports have noted that detailed anatomical or activation mapping of FVPs is inherently challenging because these tracts lack a discrete, targetable ventricular insertion and are closely integrated with the His-Purkinje system.^1^^,^^2^^,^^5^ Accordingly, the diagnosis of an FVP relies primarily on characteristic electrophysiological findings rather than on localization by mapping. Given the limited diagnostic yield and the absence of therapeutic implications, detailed mapping was not attempted in the present case.

Surface electrocardiographic findings in FVP are often subtle and nonspecific. A short PR interval with minimal preexcitation, near-normal QRS duration, and an isoelectric QRS complex in lead V_1_ with small positive delta waves in the lateral precordial leads have been described and may closely resemble septal accessory pathways.^1^^,^^2^ This overlap frequently leads to misdiagnosis and unnecessary concern for malignant arrhythmias associated with WPW syndrome.

From an electrophysiological perspective, a fixed HV interval regardless of atrial pacing, junctional rhythm, or adrenergic stimulation is the hallmark feature distinguishing FVPs from atrioventricular accessory pathways.^1^^,^^2^ In the present case, the HV interval remained constant across multiple pacing maneuvers and during isoproterenol infusion, strongly arguing against the presence of an atrioventricular bypass tract.

The disappearance of ventricular preexcitation during atrial extrastimulus pacing with the emergence of complete right bundle branch block morphology reflects functional refractoriness of the right bundle branch rather than loss of accessory pathway conduction. This phenomenon has been previously described in patients with FVP and should not be misinterpreted as evidence of a septal accessory pathway.^2^

Recognition of an FVP has important clinical implications. Ablation targeting these pathways is unnecessary and potentially harmful due to the risk of injury to the His-Purkinje system and subsequent atrioventricular block.^3^^,^^6^ In our patient, slow pathway ablation was performed to address dual atrioventricular nodal physiology, whereas the FVP was appropriately left untreated, resulting in an uneventful clinical course.

This case underscores the importance of careful integration of surface electrocardiographic findings with intracardiac electrophysiological assessment to correctly identify benign causes of ventricular preexcitation and avoid inappropriate ablation.

## Funding Support and Author Disclosures

The authors have reported that they have no relationships relevant to the contents of this paper to disclose.
